# Clinical use of the SelectMDx urinary-biomarker test with or without mpMRI in prostate cancer diagnosis: a prospective, multicenter study in biopsy-naïve men

**DOI:** 10.1038/s41391-021-00367-8

**Published:** 2021-05-03

**Authors:** Rianne J. Hendriks, Marloes M. G. van der Leest, Bas Israël, Gerjon Hannink, Anglita YantiSetiasti, Erik B. Cornel, Christina A. Hulsbergen-van de Kaa, O. Sjoerd Klaver, J. P. Michiel Sedelaar, Wim Van Criekinge, Hans de Jong, Peter F. A. Mulders, E. David Crawford, Jeroen Veltman, Jack A. Schalken, Jelle O. Barentsz, Inge M. van Oort

**Affiliations:** 1 Department of Urology, Radboud University Medical Center, Nijmegen, The Netherlands; 2 Department of Radiology and Nuclear medicine, Radboud University Medical Center, Nijmegen, The Netherlands; 3 Department of Operating Rooms, Radboud Institute for Health Sciences, Nijmegen, The Netherlands; 4 Department of Urology, Ziekenhuis Groep Twente, Hengelo, The Netherlands; 5 Laboratory Pathology Oost-Nederland, Hengelo, The Netherlands; 6 Department of Urology, Maasstad Hospital, Rotterdam, The Netherlands; 7 Department of Data Analysis and Mathematical Modelling, Ghent University, Ghent, Belgium; 8 MDxHealth B.V, Nijmegen, The Netherlands; 9 Department of Urology, University of California San Diego, San Diego, CA USA; 10 Department of Radiology, Ziekenhuis Groep Twente, Hengelo, The Netherlands

**Keywords:** Diagnostic markers, Prostate cancer, Diagnostic markers, Prostate cancer

## Abstract

**Background:**

Risk stratification in men with suspicion of prostate cancer (PCa) requires reliable diagnostic tests, not only to identify high-grade PCa, also to minimize the overdetection of low-grade PCa, and reduction of “unnecessary” prostate MRIs and biopsies. This study aimed to evaluate the SelectMDx test to detect high-grade PCa in biopsy-naïve men. Subsequently, to assess combinations of SelectMDx test and multi-parametric (mp) MRI and its potential impact on patient selection for prostate biopsy.

**Methods:**

This prospective multicenter diagnostic study included 599 biopsy-naïve patients with prostate-specific antigen level ≥3 ng/ml. All patients underwent a SelectMDx test and mpMRI before systematic transrectal ultrasound-guided biopsy (TRUSGB). Patients with a suspicious mpMRI also had an in-bore MR-guided biopsy (MRGB). Histopathologic outcome of TRUSGB and MRGB was used as reference standard. High-grade PCa was defined as ISUP Grade Group (GG) ≥ 2. The primary outcome was the detection rates of low- and high-grade PCa and number of biopsies avoided in four strategies, i.e., (1) SelectMDx test-only, (2) mpMRI-only, (3) SelectMDx test followed by mpMRI when SelectMDx test was positive (conditional strategy), and (4) SelectMDx test and mpMRI in all (joint strategy). A positive SelectMDx test outcome was a risk score of ≥−2.8. Decision curve analysis (DCA) was performed to assess clinical utility.

**Results:**

Prevalence of high-grade PCa was 31% (183/599). Thirty-eight percent (227/599) of patients had negative SelectMDx test in whom biopsy could be avoided. Low-grade PCa was not detected in 35% (48/138) with missing 10% (18/183) high-grade PCa. Yet, mpMRI-only could avoid 49% of biopsies, not detecting 4.9% (9/183) of high-grade PCa. The conditional strategy reduces the number of mpMRIs by 38% (227/599), avoiding biopsy in 60% (357/599) and missing 13% (24/183) high-grade PCa. Low-grade PCa was not detected in 58% (80/138). DCA showed the highest net benefit for the mpMRI-only strategy, followed by the conditional strategy at-risk thresholds >10%.

**Conclusions:**

SelectMDx test as a risk stratification tool for biopsy-naïve men avoids unnecessary biopsies in 38%, minimizes low-grade PCa detection, and misses only 10% high-grade PCa. Yet, using mpMRI in all patients had the highest net benefit, avoiding biopsy in 49% and missing 4.9% of high-risk PCa. However, if mpMRI availability is limited or expensive, using mpMRI-only in SelectMDx test positive patients is a good alternative strategy.

## Introduction

The diagnostic evaluation of men with clinical suspicion of prostate cancer (PCa) remains challenging. Suspicion of PCa is based on serum prostate-specific antigen (PSA) levels and/or abnormal digital rectal examination (DRE). The definitive diagnosis depends on prostate tissue histopathology obtained by biopsy [1]. However, absence of reliable strategies discriminating high-grade PCa from no or low-grade PCa leads to overdiagnosis (and potentially overtreatment) of indolent disease and unnecessary biopsy-related morbidity [2]. Various diagnostic tools, including imaging, urine- and blood-based biomarkers, and risk prediction models, are developed to aid in the decision of whether a biopsy is presumed necessary [3–7]. Recent clinical trials have supported the concept of incorporating molecular markers into the evaluation algorithm to identify men who would benefit from diagnosis and treatment [7, 8]. The most recent oncologic guidelines on PCa recommend multi-parametric magnetic resonance imaging (mpMRI) before prostate biopsy [9, 10]. However, this approach of pre-biopsy imaging is MRI-quality-dependent (i.e., quality of images and readers), costly and its availability is limited in some regions [11]. Alternatively, urine- and blood-based biomarkers have shown promising performances in predicting high-grade PCa [12–16]. These biomarkers provide an objective test result compared to reader-dependent mpMRI-outcomes, and urine/blood samples can be obtained in routine urology practice.

The SelectMDx test is a urine-based molecular test in which mRNA expression levels of HOXC6 and DLX1 are combined with clinical risk factors. The SelectMDx test demonstrated high sensitivity for the detection of high-grade PCa and can be used to select men who should undergo mpMRI and/or prostate biopsy [12, 17–19]. The aim of this prospective, multicenter study was to evaluate the clinical performance of the SelectMDx test and in combination with mpMRI for the detection of high-grade PCa in biopsy-naïve men with an elevated PSA level with an optimized biopsy reference outcome, that is, combined results of transrectal ultrasound-guided biopsy (TRUSGB) with subsequently MR in-bore guided biopsy (MRGB).

## Material and methods

### Study population

**Fig. 1:**
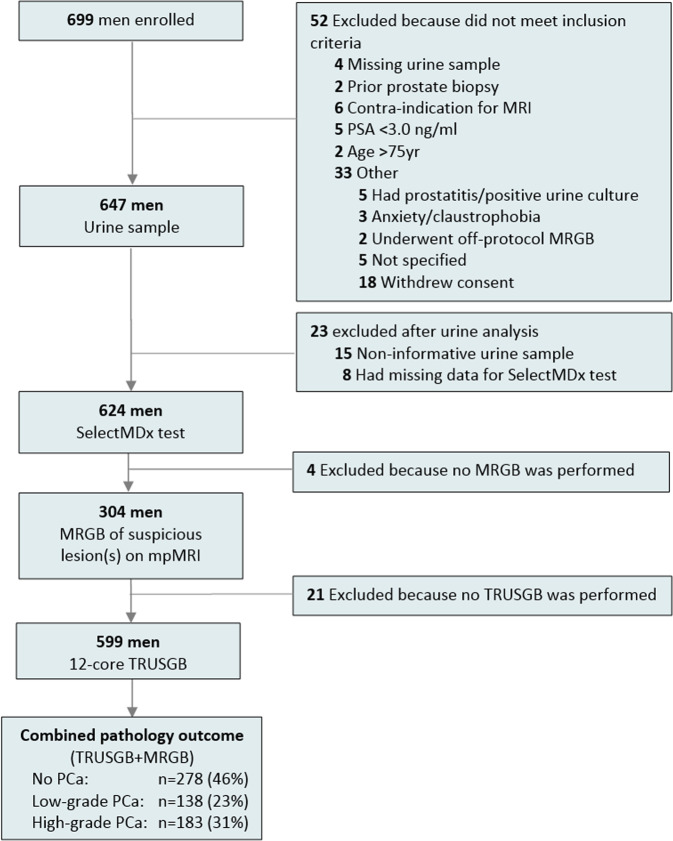
Study Flow Diagram.

In this previously described prospective, multicenter study, 699 prostate biopsy-naïve patients aged 50–75 years with a PSA level of ≥3.0 ng/ml were referred by general practitioners and enrolled by urologists in four hospitals in the Netherlands (one university hospital and three teaching hospitals) (Fig. 1) [6]. All subjects gave their written informed consent. The study protocol was approved by the Institutional Review Boards and registered in the Netherlands Trial Register (identifier NTR5555).

### The SelectMDx test

After inclusion, a first-voided urine sample was collected from each participant after DRE and used to determine the SelectMDx risk score (MDxHealth B.V., Nijmegen, The Netherlands). This SelectMDx test outcome is a risk score in which mRNA expression levels from HOXC6 and DLX1 and clinical risk factors: age, DRE result, PSA, and prostate volume (measured with TRUS) are combined. The result is a continuous risk score of −6 to 6 (higher scores reflect a higher risk for the presence of high-grade PCa), which is converted to the percent likelihood of identifying high-grade PCa in a subsequent biopsy. The cut-off for a positive SelectMDx test is a risk score −2.8. This value corresponds with a percent likelihood of 13% that subsequent biopsy would identify high-grade PCa [18, 19]. The SelectMDx test results were blinded to the biopsy outcomes.

### Multi-parametric MRI and biopsy procedure

All subjects underwent a mpMRI performed at a 3-Tesla MRI scanner (Magnetom Skyra, Siemens Healthineers, Erlangen, Germany). Two independent central radiologists interpreted images according to the Prostate Imaging Reporting and Data System version 2 (PI-RADS) [20, 21]. Disagreements in PI-RADS scores were resolved by consensus. Lesions scored PI-RADS 3–5 were considered suspicious for high-grade PCa, and subsequently, MRGB (2–4 cores per suspicious lesion) was performed. In case of multiple suspicious lesions on mpMRI, the highest PI-RADS score lesion was used as index lesion. Finally, all participating patients underwent standard systematic 12-core TRUSGB. TRUSGB was performed by clinicians blinded to the mpMRI results.

### Histopathological evaluation

A specialized genitourinary pathologist centrally reviewed the biopsies. Histological grading was assessed according to the 2014 International Society of Urological Pathology grading system (ISUP Grade groups; GG) [22]. High-grade PCa was defined as GG ≥ 2 and low-grade PCa as GG1. The outcome of the combined biopsies (TRUSGB and MRGB) was used as a reference standard.

### Statistical analysis

**Fig. 2:**
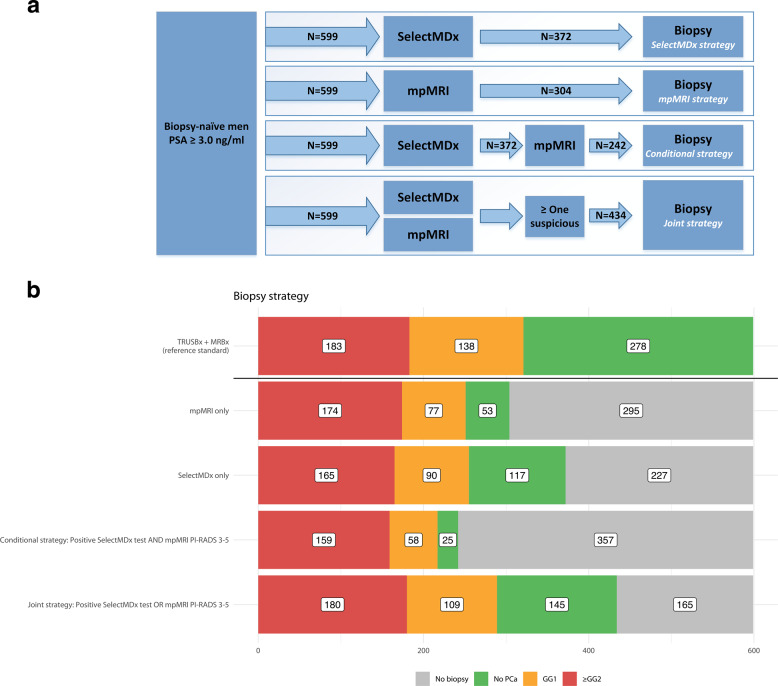
Diagnostic Strategies and PCa Detection Rate. **a** Diagnostic strategies for the detection of high-grade PCa assessed by the SelectMDx test with or without mpMRI. **b** PCa detection rate and performed biopsies for the diagnostic strategies.

Statistical analyses were performed with SPSS version 25.0 (IBM Corp., Armonk, NY, USA) and R version 3.6.3 (R Foundation for Statistical Computing, Vienna, Austria). Characteristics were described using medians and interquartile ranges (IQR) for continuous variables and as numbers with percentages for categorical variables. The following strategies of combining the SelectMDx test and mpMRI for detection of high-grade PCa and its impact on biopsy decision were assessed (Fig. 2a):

(1) SelectMDx-only strategy: SelectMDx test for everyone, then biopsy anyone with a positive result.

(2) mpMRI-only strategy: mpMRI for everyone, then biopsy anyone with a suspicious mpMRI (i.e., PI-RADS 3-5).

(3) Conditional strategy: SelectMDx test for everyone, followed by mpMRI-only for those with a positive SelectMDx test. Biopsy those with a suspicious mpMRI.

(4) Joint strategy: SelectMDx test and mpMRI for everyone, then biopsy anyone who has either a positive SelectMDx test or a suspicious mpMRI.

Clinical usefulness of different diagnostic strategies was evaluated by decision curve analysis (DCA) [23, 24]. DCA determines the advantage (net benefit) of a prediction model by examining the theoretical relationship between the threshold probability of an event (i.e., high-grade PCa upon biopsy) and the relative value of false-positive and false-negative results.

## Results

### Study population

After exclusion of 100 men, 599 patients were included in this study (see Fig. 1). Patient and diagnostic characteristics are summarized in Table 1. The median age of the participants was 65 years (IQR 59–68) and the PSA level 6.4 ng/ml (IQR 5.0/8.7). The overall PCa detection rate was 321/599 (54%); 138/599 (23%) had low-grade PCa (GG1) and 31% (183/599) had high-grade PCa (GG ≥ 2).

**Table 1:** Patient and diagnostic characteristics.

Variable	Total cohort, No. (%)		No PCa, No. (%)		Low-grade PCa, No. (%)		High-grade PCa, No. (%)	
No.	599	(100)	278	(46)	138	(23)	183	(31)
Age, yrs, median, (IQR)	65	(59–68)	63	(58–67)	64	(59–68)	66	(63–70)
Digital rectal exam								
Suspicious	166	(28)	47	(17)	30	(22)	89	(49)
Normal	433	(72)	231	(83)				
Prostate volume on TRUS, ml, median, (IQR)	50	(36–68)	58	(42–76)	50	(35–66)	42	(30–56)
Family history for PCa								
Positive	112	(19)	44	(16)	23	(17)	45	(25)
Negative	487	(81)	234	(84)	115	(83)	138	(75)
PSA, ng/ml, median, (IQR)	6.4	(5.0–8.7)	5.9	(4.8–7.8)	6.2	(4.9–7.3)	7.5	(5.7–12)
SelectMDx test								
Positive	372	(62)	117	(42)	90	(65)	165	(90)
Negative	227	(38)	161	(58)	48	(35)	18	(10)
mpMRI								
PI-RADS 1–2	295	(49)	225	(81)	61	(44)	9	(5)
PI-RADS 3	38	(6)	18	(6)	11	(8)	9	(5)
PI-RADS 4	129	(22)	30	(11)	41	(30)	58	(32)
PI-RADS 5	137	(23)	5	(2)	25	(18)	107	(58)

*Yrs* years, *PCa* prostate cancer, *GG* Gleason grade, *DRE* digital rectal exam, *TRUS* transrectal ultrasound, *PSA* prostate-specific antigen, *mpMRI* multi-parametric magnetic resonance imaging, *PI-RADS* Prostate Imaging Reporting and Data System, *IQR* interquartile range.

### Diagnostic performance of the SelectMDx test

**Fig. 3:**
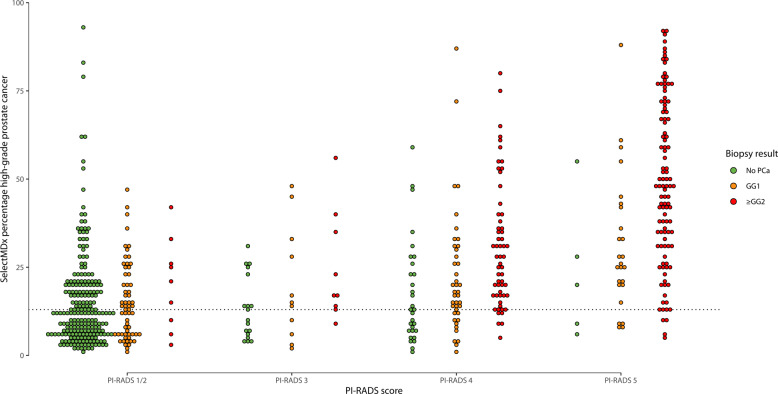
Select MDx test, histopathology and PI-RADS categories.

**Fig. 4:**
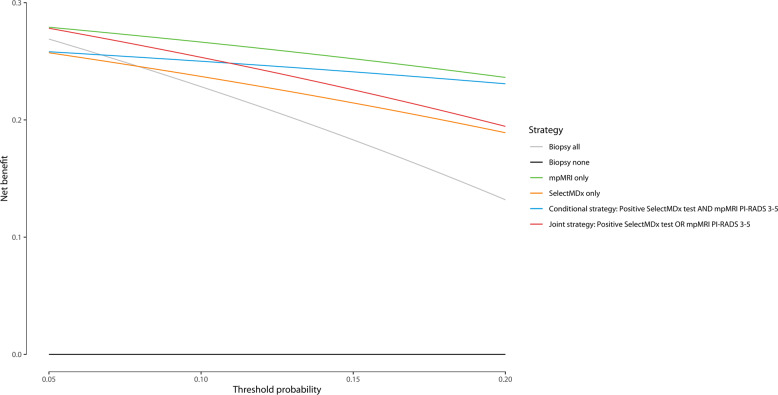
Decision curve analysis for the diagnostic strategies for the detection of high-grade PCa. Decision curve analysis for the detection of high-grade PCa of the SelectMDx-test-only, mpMRI-only, and with a positive SelectMDx test and/or suspicious mpMRI. Decision curve analysis using threshold probabilities ranging from 5 to 20% for the four diagnostic strategies. The strategy with the highest net benefit regarding high-grade PCa at a specific threshold probability is clinically most useful.

Figure 3 shows the SelectMDx test results and the combined histopathology outcome in the PI-RADS categories. Using the SelectMDx cut-off value −2.8, 38% (227/599) of the patients had a negative SelectMDx test. Of these, 71% (161/227) did not have PCa and 21% (48/227) had low-grade PCa (GG1). In 90% (165/183) of patients with high-grade PCa, the SelectMDx test was positive. Eighteen high-grade cancers were missed (10 × GG2; 6 × GG3; 2 × GG4). Sixty-two percent (372/599) of patients had a positive SelectMDx test, of whom 44% (165/372) had GG ≥ 2, 24% (90/372) a GG1, and 31% (117/372) no PCa. DCA showed that the SelectMDx test had a higher net benefit compared to biopsy all men to detect high-grade PCa, at-risk thresholds 7.9–20% (Fig. 4).

### Clinical performance of different strategies using the SelectMDx test and/or mpMRI

With mpMRI alone (i.e., perform prostate biopsy in all men with a suspicious mpMRI: PI-RADS 3-5) 95% (174/183) of high-grade PCa was detected. Nine high-grade PCa cases remained undetected (8 × GG2; 1 × GG3). Forty-four percent (61/138) of low-grade PCa was not detected with a biopsy avoidance of 49% (295/599). Strategies of combining the SelectMDx test with mpMRI for detection of high-grade PCa and its impact on biopsy decisions are presented in Table 2 and Fig. 2a, b. Using the conditional strategy with, restricting biopsy to patients with both tests positive/suspicious, reduces the number of men undergoing mpMRI by 38% (227/599), the number of biopsies by 60% (357/599), and overdetection of low-grade PCa by 58% (80/138), at the cost of missing 13% (24/183) high-grade PCa (16 × GG2; 6 × GG3; 2 × GG4). The joint strategy of performing a biopsy in men with a positive SelectMDx test or a suspicious mpMRI yielded the highest detection rate of high-grade PCa among the different strategies: 98% (180/183) of high-grade PCa was detected, with a reduction in detection of low-grade PCa of 21% (29/138) and a 28% (165/599) reduction of biopsies. Only three cases of high-grade PCa were missed (2 × GG2; 1 × GG3). The negative predictive values (NPV) of the SelectMDx test-only, mpMRI-only, the conditional and joint strategies were 92%, 97%, 93%, and 98%, respectively, and the reduction of low-grade PCa overdetection for the four strategies was 35%, 44%, 58%, and 21%, respectively. DCA showed the highest net benefit for the mpMRI-only strategy. The conditional strategy with a positive SelectMDx test followed by a suspicious mpMRI had the second-highest net benefit at-risk thresholds 11–20% (Fig. 3).

**Table 2:** Impact of the diagnostic strategies.

Strategy	Biopsy avoidance, % (No.)		mpMRI avoidance, % (No.)		Reduction low-grade PCa, % (No.)		High-grade PCa detection, % (No.)		NPV, % (95% CI)	PPV, % (95% CI)	Se, % (95% CI)	Sp, % (95% CI)
Biopsy all	NA		NA		NA		100	(183)	NA	NA	NA	NA
SelectMDx	38	(227)	100	(599)	35	(48)	90	(165)	92 0.88–0.95	44 0.39–0.50	90 0.85–0.94	50 0.45–0.55
mpMRI	49	(295)	0	(0)	44	(61)	95	(174)	97 0.94–0.99	57 0.51–0.63	95 0.91–0.98	69 0.64–0.73
Conditional	60	(357)	38	(227)	58	(80)	87	(159)	93 0.90–0.96	66 0.59–0.72	87 0.81–0.91	80 0.76–0.84
Joint	28	(165)	0	(0)	21	(29)	98	(180)	98 0.95–0.99	41 0.37–0.46	98 0.95–0.99	39 0.34–0.44

*NA* not applicable, *mpMRI* multi-parametric magnetic resonance imaging, *PCa* prostate cancer, *NPV* negative predictive value, *PPV* positive predictive value, *Se* sensitivity, *Sp* specificity.

### SelectMDx test in cases with mpMRI outcome PI-RADS 3

In total, 6% (38/599) of patients had a PI-RADS 3 mpMRI outcome, 76% (29/38) of these men did not have PCa or had GG1, and 24% (9/38) of patients had high-grade PCa. If biopsy in this group would not be performed in patients with a negative SelectMDx test (*n* = 16), the reduction of unnecessary biopsies would be 42% (16/38), at the risk of missing high-grade PCa in two men only (1 × GG2; 1 × GG3).

## Discussion

The major challenge in PCa diagnosis is to develop a diagnostic pathway that reduces the number of unnecessary biopsy procedures and minimizes overdetection of low-grade PCa but also has a high yield in detecting high-grade PCa. Currently, the American and European Urological Associations recommend mpMRI before prostate biopsy [9]. Following these recent guidelines, DCA in our study showed mpMRI in all patients before prostate biopsy to be the strategy with the highest net benefit. Yet, accessibility to high-quality mpMRI and adequate PI-RADS interpretation could be limited and expensive [11, 25, 26]. Therefore, other more easily to execute, affordable, and reliable tests are needed. Either as an alternative for mpMRI or as a stratification tool to optimize efficient use of available mpMRI capacity. This is the first prospective, multicenter study in biopsy-naïve men that combined the SelectMDx test, mpMRI and used histopathology outcome of TRUSGB and MRGB as reference standard, because of the known risk of undergrading or missing high-grade PCa with TRUSGB alone [26].

To improve the evaluation of patients with suspicion of PCa and to overcome limited mpMRI availability, molecular biomarkers can be integrated into the work-up. The SelectMDx test is such a molecular urinary-biomarker test that is easy to execute and shows high sample validity [27]. In our study, 98% of urine samples passed quality control and gave a valid result, confirming the high informative rate (95 and 96%) reported in the study of Van Neste et al. [12].

With a positive SelectMDx test, the detection rate of high-grade PCa was 44%. Restricting biopsy to patients with a positive SelectMDx test could reduce over one-third of biopsies, at the cost of missing 10% (18/183) high-grade PCa, including 10 × GG2, 6 × GG3, and 2 × GG4. The sensitivity of 90% is similar to previous studies by Van Neste et al. and Haese et al. [12, 19]. This shows the validity of this promising objective test opposed to the reader-dependent mpMRI [28].

In this study, DCA showed the highest net benefit for the mpMRI strategy, and the conditional strategy was second-best with a risk threshold 11–20%. When a positive SelectMDx test was combined with a suspicious mpMRI (conditional strategy), 60% of biopsies could be avoided. This biopsy avoidance rate is higher compared to the mpMRI strategy (49%) in our study and also compared to recent studies for mpMRI that showed biopsy avoidance rates of 33% (95% confidence interval: 26–41%) [29]. With the conditional strategy, 24 high-grade cancers were missed (16 × GG2 and 8 × GG ≥ 3), compared to eight GG2 and one GG3 PCa when performing only mpMRI in all patients. However, the conditional strategy could still be the preferred approach when there is limited availability of mpMRI, quality is of nonexpert level or because of financial reasons because mpMRI is more expensive than the SelectMDx test. Another important finding was that the overdetection of indolent, low-grade PCa could be reduced by 21% with the joint strategy and even up to 58% when using the conditional strategy.

There are some limitations to address. First, as reported in previous studies, the SelectMDx test results were based on a standard cut-off value of ≥13% (≥−2.8) [18, 19]. However, this cut-off value depends on the balance between detection of high-grade PCa, biopsy avoidance, and overdetection of low-grade PCa. To accommodate a critical appraisal by clinicians of the balance of saving biopsies and missing high-grade PCa and to provide a personalized interpretation of the SelectMDx test outcomes in shared decision-making, other cut-off values for a positive SelectMDx test could be considered. In Table S1 (Supplementary material), various cut-off values for a positive SelectMDx test were applied to a standardized number of 1000 men to assess the detection rate of high-grade/low-grade PCa and the number of biopsies avoided including sensitivity, specificity, NPV, and PPV. From eTable 1, a personalized strategy can be chosen that includes the potential risk of missing high-grade PCa. Second, high-grade tumors might have been missed by both systematic biopsy and targeted in-bore biopsy. Whole-mount prostatectomy would be a better gold standard. This is not possible, as it is unethical to perform prostatectomy in patients that do not have cancer. An alternative approach is using template prostate mapping biopsy as a reference standard; however, this a highly invasive technique. Third, we evaluated various diagnostic strategies for the detection of high-grade PCa. However, the optimal strategy depends on many factors, such as urologists’ and patients’ preference, that is, the risk of unnecessary biopsy and overdiagnosis of low-grade PCa versus the risk of missing (or delayed detection of) high-grade PCa. Furthermore, it is important to be aware of the local access to resources such as the availability and quality of prostate mpMRI. Fourth, our mpMRI images were evaluated by experienced radiologists, strictly according to PI-RADS v2 recommendations. It is challenging to reproduce these optimal conditions into standard practice. On the other hand, in our previous study, we showed a positive outcome of a training program that improved inter-reader agreement and biopsy decisions [6].

In the future, a head-to-head comparison with other biomarker tests (e.g., phi, 4K score) and risk calculators should be executed to determine the best combination of these easily implementable diagnostic tools [13–15]. Moreover, in the subgroup of PI-RADS 3 (“uncertain”) mpMRI outcomes, the SelectMDx test could be especially helpful, with a biopsy avoidance rate of 42% (16/38) in our study. Furthermore, the SelectMDx test could be of guidance in the PSA “gray area” (PSA 3–10 ng/ml), with a previously published NPV of 95% [19]. In this cohort, 84% (506/599) of patients had a PSA value of 3–10 ng/ml. The diagnostic performance of the SelectMDx test remained almost equal in this selected subgroup (Table S2, Supplementary material), confirmation of these findings is needed. Finally, the cost-effectiveness of implementing the SelectMDx test in daily practice (with or without mpMRI) should be subject to future studies. In previous reports, the strategy of performing the SelectMDx test to select patients for biopsy was beneficial in terms of costs compared to PSA testing alone [30–32]. However, this needs to be re-evaluated integrating mpMRI in the diagnostic pathway, in terms of applying a balance between avoiding mpMRIs and biopsies, reducing overdiagnosis and missing, or delaying, the detection of high-grade PCa. The cost of high-quality mpMRI plays an important role in cost-effectiveness. In other words, even though the mpMRI strategy shows the highest net benefit, a conditional strategy could be the optimal approach in regions where mpMRI of adequate quality is expensive. Finally, for individual patients the “optimal” clinical diagnostic strategy should be established with shared decision-making and is determined by clinical parameters and patients’ as well as clinicians’ and health care preferences.

To conclude, the SelectMDx test can be used in the detection of PCa as a risk stratification tool for biopsy-naïve men to minimize the detection of low-grade PCa and the risk of missing high-grade PCa while avoiding unnecessary prostate biopsies. The use of the SelectMDx test resulted in a 38% reduction of biopsy procedures, a 35% reduction of overdetection of low-grade PCa and could save 38% of mpMRIs, at the cost of missing 10% of high-grade PCa compared to biopsy all patients. Yet, the use of mpMRI in all patients to select for prostate biopsy has the highest net benefit as a pre-biopsy stratification tool. However, especially when mpMRI availability is limited or costly, the SelectMDx test could be used to select patients at risk for high-grade PCa for further diagnostics.

## Supplementary information

Supplemental material
