# Supplementary material for: Clinical use of the SelectMDx urinary-biomarker test with or without mpMRI in prostate cancer diagnosis: a prospective, multicenter study in biopsy-naïve men
Source: Prostate Cancer Prostatic Dis. 2021 May 3;24(4):1110–9. doi: 10.1038/s41391-021-00367-8 (PMC8616754; doi:10.1038/s41391-021-00367-8)
Supplement: Supplementary file 1 — Supplemental material [file 41391_2021_367_MOESM1_ESM.docx]

**Supplementary material**

To provide a personalized interpretation of the SelectMDx-test outcomes in shared decision-making, for example the need to lower the chance of missing high-grade cancer, the detection rates of high-grade and low-grade cancer and biopsy avoidance at different SelectMDx-test cut-off values were assessed, including sensitivity, specificity, negative predictive value (NPV) and positive predictive value (PPV).

**Table S1 - Overview of various risk cutoff values for the SelectMDx test and the number of biopsies saved and low-grade/high-grade PCa missed in a standardized number of 1000 men**

| **Cut-off value SelectMDx-test** | **No. of men biopsied**  **(A)** | **No. of biopsies saved**  **(B)** | | **No. low-grade PCa detected**  **(% of A)** | | **No. low-grade PCa missed**  **(%B)** | | **No. high-grade PCa detected**  **(% of A)** | | **No. high-grade PCa missed**  **(%B)** | | **Sensitivity**  **(CI)**** | **Specificity**  **(CI)**** | **PPV**  **(CI)**** | **NPV**  **(CI)**** |
| --- | --- | --- | --- | --- | --- | --- | --- | --- | --- | --- | --- | --- | --- | --- | --- |
| Total | 1000 | NA | NA | 230 | 0.23 | NA | NA | 306 | 0.31 | NA | NA | 1.0 | 0 | 0.31 | NA |
| 5% | 905 | 95 | 0.10 | 205 | 0.23 | 25 | 0.26 | 304 | 0.34 | 2 | 0.02 | 0.99  (0.97-1.00) | 0.13  (0.10-0.17) | 0.34  (0.30-0.38) | 0.98  (0.91-1.00) |
| 10% | 723 | 277 | 0.28 | 169 | 0.23 | 62 | 0.22 | 292 | 0.40 | 13 | 0.05 | 0.96  (0.92-0.96) | 0.38  (0.33-0.43) | 0.40  (0.36-0.45) | 0.95  (0.91-0.98) |
| 13%***** | 621 | 379 | 0.38 | 150 | 0.24 | 80 | 0.21 | 275 | 0.44 | 30 | 0.08 | 0.90  (0.85-0.94) | 0.50  (0.45-0.55) | 0.44  (0.39-0.50) | 0.92  (0.88-0.95) |
| 15% | 579 | 421 | 0.42 | 134 | 0.23 | 97 | 0.23 | 265 | 0.46 | 40 | 0.10 | 0.87  (0.81-0.91) | 0.55  (0.50-0.60) | 0.46  (0.40-0.51) | 0.90  (0.86-0.94) |
| 20% | 481 | 519 | 0.52 | 104 | 0.22 | 127 | 0.24 | 244 | 0.51 | 62 | 0.12 | 0.80  (0.73-0.85) | 0.66  (0.61-0.70) | 0.51  (0.54-0.57) | 0.88  (0.84-0.91) |
| **Cut-off value corresponding with the SelectMDx test value of ≥-2.8*  *** CI represents 95% confidence interval of n=599*  *CI= 95% confidence interval PCa= prostate cancer. Se=Sensitivity, Sp=specificity, PPV=positive predictive value, NPV=negative predictive value* | | | | | | | | | | | | | | | |

**Table S2 - Diagnostic performance of the SelectMDx test for different thresholds for men with a PSA 3-10 ng/ml (n=506)**

| **Cut-off value SelectMDx-test** | **Sensitivity**  **(CI)**** | **Specificity**  **(CI)**** | **PPV**  **(CI)**** | **NPV**  **(CI)**** |
| --- | --- | --- | --- | --- |
| Total | 1.0  (0.097-1.0) | 0 | 0.25  (0.21-0.29) | NA |
| 5% | 0.99  (0.96-1.0) | 0.14  (0.11-0.18) | 0.28  (0.24-0.32) | 0.98  (0.90-1.00) |
| 10% | 0.94  (0.88-0.97) | 0.40  (0.35-0.45) | 0.34  (0.29-0.39) | 0.95  (0.90-0.98) |
| 13%* | 0.86  (0.79-0.91) | 0.52  (0.47-0.57) | 0.37  (0.32-0.43) | 0.92  (0.87-0.95) |
| 15% | 0.81  (0.73-0.88) | 0.57  (0.52-0.62) | 0.39  (0.33-0.45) | 0.90  (0.85-0.93) |
| 20% | 0.72  (0.64-0.80) | 0.69  (0.64-0.73) | 0.44  (0.37-0.51) | 0.88  (0.84-0.92) |
| **Cut-off value corresponding with the SelectMDx test value of ≥-2.8*  *** CI represents 95% confidence interval of n=599*  *CI= 95% confidence interval PCa= prostate cancer. Se=Sensitivity, Sp=specificity, PPV=positive predictive value, NPV=negative predictive value* | | | | |
